# Hospital resource use and costs in autoimmune encephalitis: a single-center retrospective cohort study

**DOI:** 10.1186/s42466-026-00491-7

**Published:** 2026-04-17

**Authors:** Niklas Keller, Dominic Bertram, Hajo Hamer, Arnd Doerfler, Stefan Schwab, Veit Rothhammer, Thanos Tsaktanis

**Affiliations:** 1 Department of Neurology, University Hospital Erlangen, Friedrich-Alexander-Universität Erlangen-Nürnberg (FAU), Schwabachanlage 6, 91054 Erlangen, Germany; 2 Department of Neurology, Klinikum rechts der Isar, TUM Munich, Munich, Germany; 3 Department of Neuroradiology, University Hospital Erlangen, Friedrich-Alexander-Universität Erlangen-Nürnberg (FAU), Erlangen, Germany; 4 Deutsches Zentrum Immuntherapie (DZI), Universitätsklinikum Erlangen, 91054 Erlangen, Germany

## Abstract

**Background:**

Autoimmune encephalitis (AE) causes substantial but insufficiently quantified hospital resource use. We aimed to estimate direct hospital costs of acute AE care and identify factors associated with prolonged length of stay (LOS).

**Methods:**

We conducted a single-center retrospective cohort study of patients with an index admission fulfilling criteria for probable or definite AE (2011–2021). Direct hospital costs within 1 year after index admission were estimated from the UKER hospital/provider perspective by applying standardized German unit costs (price year 2020) to observed non-ICU ward days, ICU days, and neurology outpatient visits. Associations with total costs were assessed using univariable OLS regression; contributors to prolonged LOS were extracted from chart review.

**Results:**

Thirty-five patients (16 female, 46%; median age 63 years [IQR 42–73]) were included (2011–2021). CNS-targeting antibodies were detected in 74%. Median LOS was 26 days (IQR 13–38), and 11 patients (31%) required ICU care. Median direct hospital costs per patient within the first year were EUR 26,547 (USD 30,264). ICU-admitted patients incurred higher hospital costs than non-ICU patients (EUR 69,822 vs. EUR 20,234 per patient; *p* < 0.001). LOS, particularly ICU LOS, was a major cost driver. Delayed diagnosis, prolonged immunotherapy, insufficient treatment response, medical complications, and ICU admission contributed to longer LOS and higher costs. Costs did not differ between seropositive and seronegative patients, but varied by antibody subtype, with NMDA-R positivity associated with higher costs.

**Conclusion:**

AE is associated with substantial direct hospital costs, especially among ICU-admitted patients. Optimizing diagnostic workflows and timely initiation of immunotherapy may reduce LOS and resource use and improve outcomes.

**Supplementary Information:**

The online version contains supplementary material available at 10.1186/s42466-026-00491-7.

## Introduction

 With an annual incidence of 0.8 to 1.5 cases per 100,000 persons, autoimmune encephalitis (AE) is a rare disease. AE is an immune-mediated inflammatory disease of the central nervous system associated with antibodies targeting intracellular or cell-surface neuronal antigens [1–3], affecting individuals across all age groups while imposing substantial burden on individual patients, their families, as well as the healthcare system [4, 5]. For yet unknown reasons aside from improved diagnostics [6, 7] its incidence has increased over the past decades. Importantly, AE is associated with severe transient or persisting neurological deficits [3, 8] including focal neurological and psychiatric symptoms as well as generalized disturbances such as altered consciousness, epileptic seizures, movement disorders, and autonomic dysfunction. Rare diseases, AE in particular, often pose challenges to treating physicians, since symptoms may be unspecific or misleading, making AE hard to detect and oftentimes misdiagnosed, delaying the start of immunomodulatory therapies and prolonging disease duration and overall length of hospital stay (LOS) [9, 10]. AE oftentimes causes immense personal and socioeconomic burden. However, the economic aspect of AE in particular has not been described in detail.

Therefore, we aimed to quantify direct hospital costs related to acute AE management at a German university neurology center and to examine associations between costs, clinical characteristics, and outcomes.

## Methods

Patients admitted to the Department of Neurology at University Hospital Erlangen (UKER) between 2011 and 2021 with a discharge diagnosis of non-infectious encephalitis were retrospectively identified using the clinical information system. Identification was based on ICD-10-GM codes “other encephalitis, myelitis, and encephalomyelitis (G04.8)” as well as “encephalitis, myelitis and encephalomyelitis unspecified (G04.9)”.

We included cases that fulfilled the diagnostic criteria for either probable (seronegative) or definite (AB-positive) AE according to the current consensus statement [11]. Cases with a discharge diagnosis of AE that did not meet these criteria, as well as cases with different specific diagnoses, e.g. multiple sclerosis, or neuromyelitis optica spectrum disorder were excluded. Analysis was limited to patients whose diagnostic work-up and acute treatment were primarily conducted at University Hospital Erlangen (UKER). We collected all available data, including clinical characteristics, outcome measures, and health care utilization. To ensure comparability across patients, analyses of healthcare utilization and costs were restricted to a uniform observation period of one year following AE diagnosis.

Clinical data were extracted retrospectively from the electronic medical records. Initial symptoms were categorized according to the consensus definition of AE, including altered mental status, psychiatric symptoms, working memory deficits, new focal central nervous system (CNS) deficits, and new-onset seizures (not attributable to pre-existing epilepsy), with or without corresponding electroencephalography (EEG) abnormalities (Table 1) [11].

**Table 1:** Diagnostic criteria for autoimmune encephalitis

Possible AE	Definite AE
Diagnosis can be made when all three of the following criteria have been met:	
• Subacute onset of working memory deficits (short-term memory loss), altered mental status*, or psychiatric symptoms	• Criteria for possible AE fulfilled + • Detection of specific antibodies (Table 2)
• At least one of the following: - New focal CNS deficits - Seizures not explained by previous known seizure disorder - CSF pleocytosis (white blood cell count of more than five cells per mm³) - MRI features suggestive of encephalitis	
• Reasonable exclusion of alternative causes	

*Altered mental status defined as decreased or altered level of consciousness, lethargy, or personality change

†Brain MRI hyperintense signal on T2-weighted fluid-attenuated inversion recovery sequences highly restricted to one or both medial temporal lobes (limbic encephalitis), or in multifocal areas involving grey matter, white matter, or both compatible with demyelination or inflammation

Laboratory and radiological data were collected, primarily acquired during the UKER hospital stay. However, if sufficient documentation from referring hospitals was available these datasets were included equally.

Magnetic resonance imaging (MRI) findings suggestive of AE were defined by neuroradiological assessment as hyperintense signals on T2-weighted fluid-attenuated inversion recovery (FLAIR) sequences, localized to one or both medial temporal lobes, or multifocal lesions involving gray matter, white matter, or both, consistent with inflammatory CNS involvement [11–13]. Contrast enhancement was defined as gadolinium enhancement on T1-weighted images. Limbic system involvement was characterized as lesions in the medial temporal lobes, amygdala, hippocampus, insula, hypothalamus, and/or thalamus.

Serum analyses included results from neural antibody panels (Table 2). Intrathecal inflammation was defined as cerebrospinal fluid (CSF) protein > 450 mg/l, white blood cell count > 5/µl, or intrathecal IgG synthesis, indicated by either ≥ 2 oligoclonal IgG bands in CSF only (pattern 2) or ≥ 2 bands in CSF only with ≥ 1 distinct band also present in both CSF and serum (pattern 3).

**Table 2:** Neural antigen immunofluorescence assay panel (including demyelinating antibodies), as commonly initiated at UKER

Hu	Anti-Neuronal Nuclear Antibody [ANNA] type 1
Ri	ANNA-2
ANNA-3	
Yo	Purkinje Cell Cytoplasmic Antibody type 1
Tr/DNER	Delta/Notch-like EGF-related receptor
Ma/Ta	
GAD65	Glutamic Acid Decarboxylase 65
Amphiphysin	
Aquaporin-4	
MOG	Myelin Oligodendrocyte Glycoprotein
Glutamate receptors • NMDAR • AMPAR	N-methyl-D-aspartate Receptor α-Amino-3-hydroxy-5-methyl-4-isoxazolepropionic acid receptor
GABAA/B receptor	Gamma-Aminobutyric Acid Receptor Type A/B
LGI1	Leucine-Rich Glioma-Inactivated Protein 1
CASPR2	Contactin-Associated Protein-like 2
IgLON5	Immunoglobulin-like Cell Adhesion Molecule 5
ZIC4	Zinc Finger Protein of the Cerebellum 4
DPPX	Dipeptidyl-Peptidase-like Protein 6
Myelin	

Abnormal EEG findings were defined as generalized or focal slowing, epileptiform discharges, seizures, or status epilepticus.

To assess morbidity, modified Rankin Scale (mRS) scores were retrospectively estimated based on clinical documentation by treating physicians and therapists. Measures of health care utilization included hospitalizations and LOS, number of outpatient visits at the Department of Neurology at UKER, and immunotherapy administered within the first year after index admission.

Contributors to prolonged LOS were identified by reviewing clinical information from all index hospitalizations exceeding 7 days.

Medical costs are commonly categorized into direct medical costs (e.g. medication, hospitalization, rehabilitation), direct non-medical costs (e.g. informal care, alternative therapies), and indirect costs (e.g. productivity losses). Direct medical costs were estimated from a hospital/provider perspective, restricted to costs incurred at University Hospital Erlangen (UKER), by applying published standardized unit costs to observed resource use (ward and ICU length of stay and outpatient visits), in line with German recommendations for health economic evaluations. Updated standardized unit costs for inpatient care (non-observation ward: 1,011.72 EUR/day, intensive care unit (ICU): 2,192.49 EUR/day), and outpatient medical care (60.66 EUR/appointment) were used for cost calculation [14–16]. Resource utilization was assessed retrospectively using data obtained from the clinical information system. Medical reports were used to retrace patients’ clinical course, collect data on LOS, ICU admissions, diagnostic procedures, as well as results, adverse events, therapies, discharge dispositions and outpatient care contacts. All costs were calculated in Euros (EUR, price year 2020; average exchange rate: EUR 1 = USD 1.14 according to the European Central Bank).

### Statistical analyses

Data were recorded in an Excel database. Statistical analysis was performed in SPSS statistics version 28.0. We compared differences in demographic, clinical, and outcome variables using the χ² test for categorical variables (i.e., binary or ordinal) and the Mann-Whitney U test for continuous variables. Univariate regression analyses were conducted, with several variables selected a priori to examine their association with total costs: sex, age, neural antibody status, total length of stay (LOS), and length of stay in the intensive care unit (ICU). Comparisons were performed using ordinary least squares (OLS) regression. Significance was set at *p* ≤ 0.05. Formal correction for multiple comparisons was not applied given the exploratory nature of the study and the relatively small sample size.

## Results

**Fig. 1:**
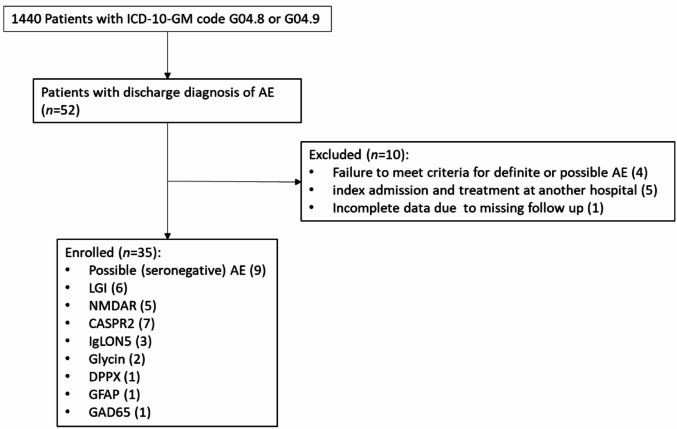
Patient selection and enrollment

Out of 52 screened admissions with a discharge diagnosis of suspected AE, 35 patients met the criteria for either definite or probable AE (Fig. 1). In 26 (74%) of these cases, autoantibodies (ABs) were detected in serum or CSF. Median age at admission was 63 (IQR 42–73) years, and 46% of patients were female. An underlying malignancy (ovarian teratoma) was identified in one patient with anti-N-methyl-D-aspartate receptor (NMDA-R) encephalitis, whereas 20% of patients had a concomitant autoimmune disorder. Three of those were under long-term immunosuppressive therapy. 

Median duration from onset to admission was 30 days. Symptoms included focal CNS deficits (11 out of 35 patients), altered mental status (26 out of 35 patients) and new-onset epileptic seizures (25 out of 35 patients) (Table 3).

**Table 3:** Patient characteristics, hospital burden and outcome measures

All patients (*n* = 35)	
Median age, y (IQR)	63 (42–73)
Female, *n* (%)	16 (46)
**Symptoms**	
Altered mental status, *n* (%)	26 (74)
New-onset seizures, *n* (%)	25 (71)
Focal CNS deficit, *n* (%)	11 (31)
Specific MRI findings, *n* (%)	20 (61)^T^
CSF inflammation, *n* (%)	22 (63)
Antibody-positive, *n* (%)	26 (74)*
Tumor, *n* (%)	1 (3)
Concomitant autoimmune disease, *n* (%)	7 (20)
Median duration from onset to admission, d (IQR)	30 (0-180)
Median total LOS, d (IQR)	26 (13–38)
Median total LOS for non-ICU admitted patients, d (IQR)	20 (11–27)
Median total LOS for ICU-admitted patients, d (IQR)	43 (32–54)
ICU – Admission, *n* (%)	11 (31)
Median mRS at admission (IQR)	3 (2–5)
Median mRS at discharge (IQR)	3 (2–5)
Deaths within 1 year, *n* (%)	1 (3)
Median hospital costs per patient, € (IQR)	26,305 (13,152 − 56,825)
Median outpatient encounters within 1 year, *n* (IQR)	4 (2–7)
Median outpatient care costs per patient, € (IQR)	243 (121–425)
Median total direct medical costs per patient, € (IQR)	26,547 (13,516 − 57,250)
Total costs, €	1,378,583

^T^MRI findings were available in 33/35 patients. Percentages refer to patients with MRI available (*n* = 33)

*Antibodies detected: NMDA-R (5), LGI1 (6), CASPR2 (7), IgLON5 (3), Glycine (2), GFAP (1), DPPX (1), GAD65 (1)

CSF analyses, including white blood cell counts and protein levels, were available for 32 of 35 patients. Evidence of CSF inflammation was found in 22 patients. CSF pleocytosis was present in 18 (51%) patients, and elevated protein levels were observed in 14 (40%) patients. Oligoclonal bands (OCB) were assessed in 28 patients, with intrathecal OCB patterns detected in 9. Neural antibody immunofluorescence testing detected distinct ABs against NMDA-R (*n* = 5), LGI1 (*n* = 6), CASPR2 (*n* = 7), IgLON5 (*n* = 3), glycine (*n* = 2), GFAP (*n* = 1), DPPX (*n* = 1), and GAD65 (*n* = 1).

MRI was available for 33 patients (94%). In 20 (61%) of these cases, MRI revealed abnormalities consistent with AE. Of those, 17 patients showed involvement of the limbic system, while 3 had extra limbic inflammatory lesions. Remaining MRI scans were either unremarkable (6/33) or showed non-specific findings, including microangiopathy or chronic ischemic lesions (7/33) (Supplementary Table 1).

Median time from index admission to initiation of immunotherapy was 6 days (IQR 3–16), with no difference between seropositive and seronegative AE.

Median index admission LOS was 26 (IQR 13–38) days. None of the patients died during the index hospitalization. One patient with LGI1-associated AE died within one year of follow-up, resulting in a nominal 1-year mortality of 3%; the suspected cause of death was pulmonary embolism. Median mRS at admission and discharge was 3 (IQR 2–5).

Median hospital costs for patients with AE were EUR 26,305 (inpatient ward and ICU costs), with no significant differences between antibody-negative and antibody-positive patients (EUR 30,352 [IQR EUR 13,152–56,235] vs. EUR 24,787 [IQR EUR 12,899–61,727], respectively). Patients were discharged to neurological rehabilitation in 19 cases; 16 patients were discharged home.

One year follow-up was available for 32 patients. Within the first year, 30 of 35 patients were readmitted at least once, with a median hospitalization count of 3 (IQR 2–4). The number of admissions did not differ between patients suffering from antibody-positive versus antibody-negative AE. Within the first year, all patients had a median of 4 outpatient visits (IQR 2–7) at UKER, leading to costs of EUR 243 (IQR EUR 121- EUR 425). In summary, median total costs per patient amounted to EUR 26,547 (IQR EUR 13,516- EUR 57,250).

Within 1 year after index admission, 31 patients received escalation immunotherapy beyond initial first-line treatment. This included rituximab (*n* = 18), intravenous immunoglobulins (IVIG; *n* = 5), cyclophosphamide (*n* = 3), mycophenolate mofetil (*n* = 2), and azathioprine (*n* = 1). One patient was treated with IVIG and rituximab within the first year. Our findings showed that seropositive AE was significantly more often treated with rituximab as second-line immunotherapy (77% AB-positive vs. 22% AB-negative; *p* = 0.003).

We compared patients admitted to ICU to patients without ICU admission (Table 4). Of the 35 patients, 11 (31%) were admitted to ICU. Reasons for ICU admission included impaired consciousness (6 out of 11 patients), status epilepticus (2 out of 11 patients) and severe medical complications, e.g., respiratory failure and consecutive need for mechanical ventilation (3 out of 11 patients). We did not detect differences between the groups regarding age, sex, AB status, or clinical presentation.

**Table 4:** Comparison between ICU admissions and non-ICU admissions

Age, median (IQR)	**Non-ICU** (*n*=24)	**ICU** (*n*=11)	*p* value
	63 (54-76)	48 (24-71)	0.174
**Female sex**, *n* (%)	10 (42)	6 (55)	0.478
**Antibody positive**, *n* (%)	18 (75)	8 (73)	0.886
**Symptoms**			
New focal CNS deficits, *n* (%)	8 (30)	4 (36)	0.670
Altered mental status, *n* (%)	17 (71)	9 (82)	0.357
New-onset seizures, *n* (%)	20 (77)	7 (64)	0.490
**MRI**, *n* (%)	17 (54)	3 (27)	0.094
**CSF inflammation**, *n* (%)	13 (54)	9 (82)	0.116
**EEG abnormalities**, *n* (%)	20 (83)	8 (73)	0.170
**LOS**, median (IQR)	20 (11.25-26.75)	43 (32-54)	**<0.001**
**mRS-Score at admission**, median (IQR)	3 (2-4)	5 (3-5)	
**mRS-Score at discharge**, median (IQR)	2 (2-3)	5 (4-5)	
**Deaths within 1 year**, *n* (%)	1 (4)	0 (0)	
**Hospital costs**, median (IQR) €	20,234 (11,382-27,063)	69,822 (56,825-101,864)	**<0.001**
**Outpatient care costs**, median (IQR) €	242 (121-409)	364 (61-424)	0.781

AE = autoimmune encephalitis; ICU = intensive care unit; IQR = interquartile range; LOS = length of stay; mRS = modified Rankin Scale

**Fig. 2:**
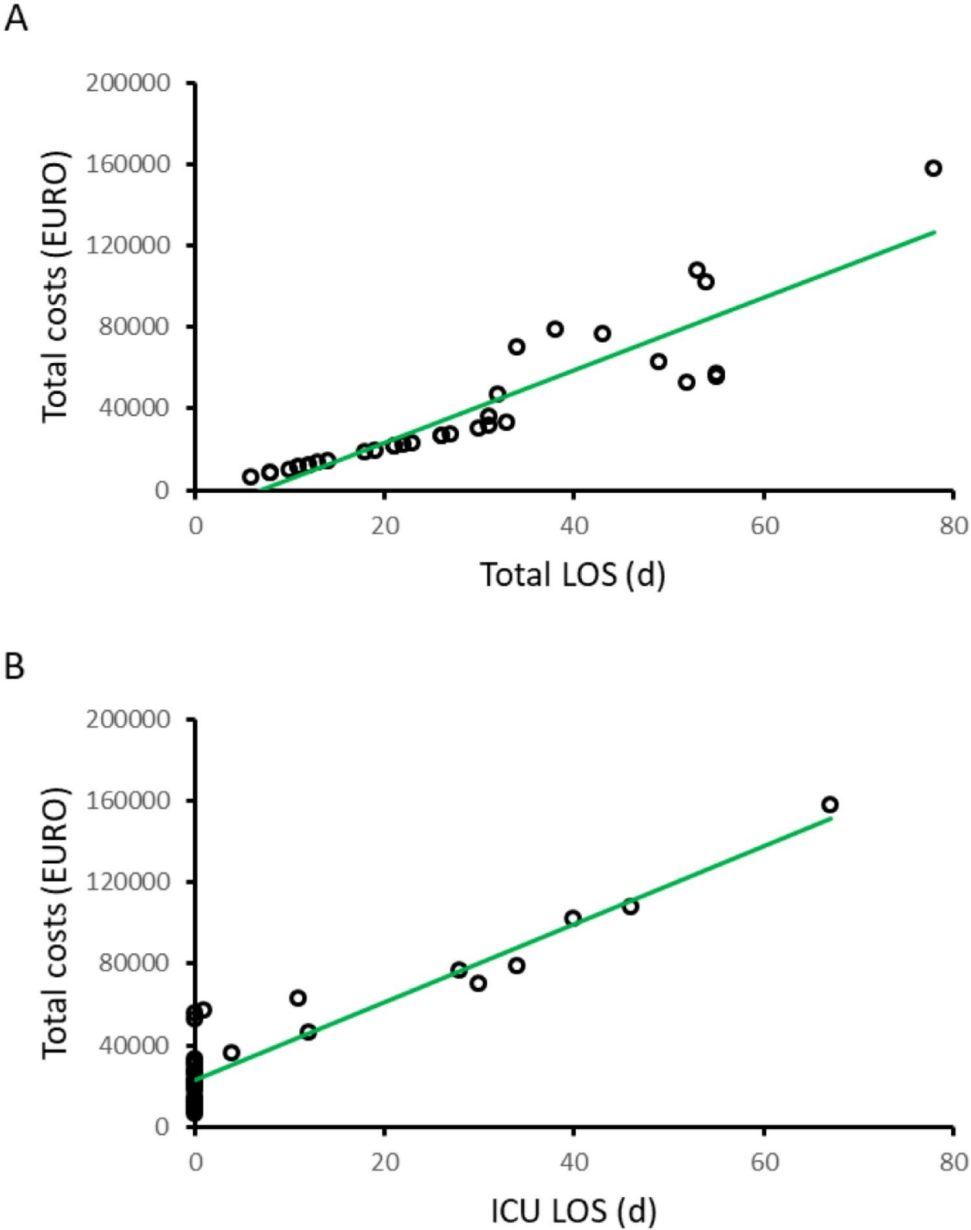
Scatterplot of hospital costs vs. (**A**) Total LOS (r2 = 0.82) vs. (**B**) ICU LOS (r2 = 0.86), lines representing linear regression LOS = Length of stay, ICU = Intensive care unit

Ordinary least squares (OLS) regression revealed that total LOS, and especially ICU LOS were strongly associated with total hospitalization costs (total LOS: *r²* = 0.82, *p* < 0.001 and ICU LOS: *r²* = 0.86, *p* < 0.001) (Fig. 2). Inclusion of age, sex, presence of cancer, or neural antibody status did not increase the proportion of explained variance. However, no significant association was identified between variables selected and outpatient care costs within the first year.

Next, we compared outcome parameters within antibody types detected in at least three patients. These analyses suggested that patients with NMDA-R antibody seropositivity exhibited longer total LOS, higher rates of ICU admissions, and higher hospitalization costs compared to those with autoimmune encephalitis associated with other antibodies (e.g., IgLON5, LGI1, CASPR2) (Supplementary Table 2). In contrast, CASPR2-associated encephalitis was associated with the shortest median LOS (12 days, [IQR 11–21]), lower rate of ICU admissions, and overall lower medical costs.

In 30 patients, index admission LOS exceeded 7 days (86%). Contributing factors included ICU admission (*n* = 9), delayed or extended diagnostic work-up (*n* = 10), prolonged immunotherapy (*n* = 19), lack of clinical response to first line therapy (*n* = 10), and adverse events (*n* = 10). Among the 19 patients receiving prolonged immunotherapy, 14 were treated with corticosteroid pulse therapy followed by plasma exchange (40%), while 5 underwent multiple plasma exchange sessions as first-line treatment (14%).

In 16 hospitalizations, multiple factors contributed to increased LOS, e.g. prolonged immunotherapy along with adverse events. In the remaining 14 cases, a single contributing factor was identified: 8 involved prolonged therapy, 6 lack of response to initial treatment, and 5 extended diagnostics. Half of the patients undergoing extended diagnostics (*n* = 3) were seronegative, two of the CASPR2 AB-positive cases received extensive EEG monitoring in the epilepsy monitoring unit. Two patients with minor deficits showed no initial response to first-line steroid pulse therapy and were discharged for observation of a potential delayed effect. After two weeks without clinical improvement, the treatment was deemed ineffective, and the patients were readmitted for second-line immunotherapy.

## Discussion

To our knowledge, this study provides one of the first detailed estimates of direct hospital-based medical costs of AE in Germany. Overall direct medical costs for acute treatment of AE comprised hospitalization and outpatient care costs and amounted to over EUR 26,000 (approximately USD 30,000). We detected differences between hospitalization costs for patients admitted to ICU (EUR 69,822, or USD 79,597) and patients without ICU admission (EUR 20,234, or USD 23,067). Costs were driven by LOS, especially in the ICU, similar to findings of a previous study in a cohort of AE patients in United States [17]. Comparable studies on AE in China and West Nile virus encephalitis in Canada revealed that costs for inpatient treatment accounted for the majority of total direct costs [18, 19]. Different proportions have been reported in a German study on cost and quality of life in patients with NMOSD and MOG-antibody associated disease, where direct medical cost accounted for less than half of the overall costs [20]. This could be explained by differences in clinical presentation, medical complications, and progression of disease [21–24].

Due to the complexity and duration of diagnostic procedures, the need for prolonged immunotherapy, and the high rate of ICU admissions, we found that patients with AE were hospitalized for extended periods frequently, resulting in high direct medical costs [25]. We identified factors contributing to longer LOS, such as delayed diagnosis and delayed start of immunotherapy, prolonged treatment, lack of response to initial immunotherapy, and adverse events.

In 10 patients, AE diagnosis was delayed due to extensive diagnostic work-up (e.g. exclusion of infectious cause, neural AB diagnostics) [26]. This cautious decision making is warranted many times and enabled definite diagnoses but prolongs initiation of treatment. Presence of specific antibodies was not associated with faster start of therapy, which could be explained by time consuming laboratory diagnostics [27–29]. Anti-neuronal antibody panels are not performed by in-house laboratory at UKER, as in most hospitals, but by an external service provider. This procedure leads to a significant prolongation of diagnosis due to the sample shipping and receipt of results. Patients with psychotic symptoms, particularly in NMDA-R encephalitis, often primarily present to psychiatrists. Misdiagnoses with primary psychosis cause a delay in AE identification and treatment initiation [30, 31]. In our cohort, 3 out of 6 patients with NMDA-R encephalitis were initially admitted to psychiatric wards. In these cases, more time was required to diagnose AE. However, the subgroup was too small for reasonable statistical analysis. In addition, in selected patients with seizures or unclear paroxysmal events, extended video-EEG monitoring or evaluation in an epilepsy monitoring unit may be clinically necessary and can contribute to longer hospital stays [32].

In AE, extended immunotherapy is also a factor contributing to prolonged hospitalization. While most patients are initially treated with steroids, approximately two thirds were treated with multiple plasmapheresis sessions every other day to achieve clinical improvement in addition. To date, literature provides no evidence for clear superiority of either steroids, plasmapheresis or IVIG in the first line therapy of AE. However, combination therapies, and most importantly, early treatment initiation are associated with better clinical outcome [33, 34]. While we did not observe any differences in the number of outpatient care encounters between antibody-positive and antibody-negative AE patients at UKER, seropositive patients more frequently received escalation immunotherapy beyond initial first-line treatment, including agents such as rituximab and IVIG.

Our study also compared the direct medical costs of different subtypes of AE. While no differences were observed between seronegative and seropositive AE overall, presence of distinct antibodies was associated with varying extent of healthcare utilization. Patients with anti-NMDA-R encephalitis were more frequently admitted to ICU, had extended LOS and consequently greater costs, than patients with anti-LGI-1 or anti-CASPR-2 encephalitis. These findings are consistent with the results of a comparable investigation conducted in China [18] and likely attributable to the typically severe course of anti-NMDA-R encephalitis, which is often characterized by psychiatric symptoms, fluctuating levels of consciousness, epileptic seizures including status epilepticus, and respiratory dysfunction, requiring sedative medication, airway protection, and mechanical ventilation [35, 36]. In contrast, CASPR2 antibody–associated AE was linked to shorter median LOS, no ICU admissions in our cohort, and consequently lower overall costs. This difference may reflect the typical clinical presentation dominated by epileptic seizures and limbic symptoms as well as the usually good response to initial treatment [37, 38]. Except for one patient requiring plasmapheresis, the remaining 6 patients suffering from anti-CASPR-2 antibodies in our cohort exhibited sufficient clinical improvement after initiation of steroid therapy.

Limitations of our study are associated with the retrospective design of the investigation. We were only able to collect data regarding direct medical costs caused by acute in hospital treatment and outpatient care at UKER. Data on rehabilitation, as well as direct non-medical costs, e.g. for domestic care or indirect cause due to loss of productivity were not recorded and therefore not evaluated. The used ICD-10-coded diagnoses to identify cases supposedly cover a broad number of patients but may not be able to identify all AE cases. In addition, patients identified through the screening process applied had to be confirmed as having AE using data from the clinical information system. LOS and resource utilization might also be increased due to comorbidities. Comorbid conditions were documented during chart review, but not systematically included in regression analyses, considering the limited sample size. Furthermore, atypical symptom onset could delay diagnosis and therefore increase total LOS, but was, for the same reason, not given consideration in statistical analyses. To avoid increasing the risk of type 2 errors, no adjustments for multiple comparisons were applied, due to the exploratory nature of our study. For outcome assessment, we had to rely on existing data. Finally, the monocentric study design and small cohort of suitable patients resulted in limitations as to the generalizability of data collected here.

In summary, treatment of patients with AE in Germany causes substantial medical resource usage and is paralleled by prolonged LOS, high rates of ICU admissions, time consuming diagnostic and treatment, and altogether high medical costs. There is a demand for measures to identify specific antibodies, but also seronegative AE, faster, to reduce delay in starting immunotherapy and hospitalization burden.

## Electronic Supplementary Material

Supplementary Material 1

## Data Availability

The data that support the findings of this study are not publicly available due to privacy and ethical restrictions involving sensitive patient information. De-identified data are available from the corresponding author upon reasonable request and subject to approval by the local ethics committee.
