# Supplementary material for: Hospital resource use and costs in autoimmune encephalitis: a single-center retrospective cohort study
Source: Neurol Res Pract. 2026 Apr 17;8(1):26. doi: 10.1186/s42466-026-00491-7 (PMC13091250; doi:10.1186/s42466-026-00491-7)
Supplement: Supplementary file 1 — Supplementary Material 1 [file 42466_2026_491_MOESM1_ESM.docx]

**Supplementary Tables**

| **Supplementary Table 1** | | | | | | | | | | | |
| --- | --- | --- | --- | --- | --- | --- | --- | --- | --- | --- | --- |
| **Patient No.** | **Age at diagnosis** | **Sex** | **Diagnosis** | **Leading symptoms** | **CSF** | **MRI**  **findings** | **Antibody titer (IFT)** | **First line immunotherapy** | **Second line immunotherapy** | **mRS**  **(at index admission)** | **mRS**  **(at discharge)** |
| 1 | 23 | f | Seronegative AE | Epileptic seizures, altered mental status | WBC: 7  Protein: 136  OCBs: - | T2 hyperintense lesion of both hippocampi | Serum: - CSF: - | Plasmapheresis | none | 2 | 2 |
| 2 | 42 | f | Seronegative AE | Epileptic seizures, altered mental status | WBC: 482  Protein: 347  OCBs: - | T2 hyperintense lesion of right hippocampus | Serum: - CSF: - | Steroid Pulse | none | 2 | 2 |
| 3 | 45 | f | Seronegative AE | Epileptic seizures, altered mental status | WBC: 3  Protein: 248  OCBs: 1 | T2 hyperintense lesion of left amygdala | Serum: - CSF: - | Steroid Pulse | Plasmapheresis | 5 | 4 |
| 4 | 69 | m | Seronegative AE | Epileptic seizures, altered mental status | WBC: 1  Protein: 394  OCBs: 1 | Limbic encephalitis on both sides | Serum: - CSF: - | Steroid Pulse | Plasmapheresis | 4 | 2 |
| 5 | 71 | m | Seronegative AE | Epileptic seizures, altered mental status | WBC: 100  Protein: 1069  OCBs: 3 | Right parietal T2 hyperintense lesion | Serum: - CSF: - | Steroid Pulse | Plasmapheresis | 2 | 2 |
| 6 | 73 | f | Seronegative AE | hemiparesis, altered mental status | WBC: 144  Protein: 636  OCBs: 4 | Gd enhancement of hippocampi, mesencephalon and pons | Serum: - CSF: - | Steroid Pulse | Plasmapheresis | 3 | 2 |
| 7 | 65 | f | Seronegative AE | Epileptic seizures, altered mental status | WBC: 7  Protein: 237  OCBs: 1 | T2 hyperintense lesion of right hippocampus | Serum: - CSF: - | Steroid Pulse | none | 2 | 2 |
| 8 | 40 | f | Seronegative AE | Epileptic seizures, altered mental status | WBC: 0  Protein: 402  OCBs: 4 | Cortical and periventricular lesions on both sides, Gd enhancement left precentral cortex | Serum: - CSF: - | Steroid Pulse | Plasmapheresis | 4 | 5 |
| 9 | 64 | f | Seronegative AE | Epileptic seizures, altered mental status | WBC: 1  Protein: 472  OCBs: 1 | Microangiopathy | Serum: - CSF: - | Plasmapheresis | none | 5 | 4 |
| 10 | 78 | m | LGI-1 AE | Cognitive impairment, epileptic seizures | WBC: 2  Protein: 446  OCBs: 4 | Gd enhancement left hippocampus | Serum: 1:320 CSF:  1:1 | Steroid Pulse | Plasmapheresis | 2 | 2 |
| 11 | 79 | f | LGI-1 AE | Faciobrachial dystonic seizures, | WBC: 0  Protein: 300  OCBs: 1 | T2 hyperintense lesion of left hippocampus | Serum: 1:320 CSF:  - | Steroid Pulse | Plasmapheresis | 4 | 3 |
| 12 | 79 | f | LGI-1 AE | Faciobrachial dystonic seizures, | WBC: 9  Protein: 384  OCB: 1 | T2 hyperintense lesion of amygdala both sides | Serum: 1:320 CSF:  - | Steroid Pulse | Plasmapheresis | 2 | 2 |
| 13 | 58 | m | LGI-1 AE | Cognitive impairment, altered mental status | WBC: 2  Protein: 326  OCBs: 1 | Increased volume of right amygdala and hippocampus | Serum: 1:640 CSF:  1:16 | Steroid Pulse | none | 3 | 3 |
| 14 | 77 | m | LGI-1 AE | Faciobrachial dystonic seizures | WBC: 1  Protein: 497  OCBs: 4 | Not available | Serum: 1:320 CSF:  - | Steroid Pulse | none | 3 | 3 |
| 15 | 63 | m | LGI-1 AE | Epileptic seizures, altered mental status | WBC: 16  Protein: 468  OCBs: 4 | T2 hyperintense lesion of right hippocampus | Serum: 1:320 CSF:  1:100 | Steroid Pulse | Plasmapheresis | 4 | 3 |
| 16 | 34 | f | NMDAR AE | Epileptic seizures, psychosis | WBC: 13  Protein: 205  OCBs: 2 | Edema of left medial temporal lobe | Serum: 1:320 CSF:  1:100 | Steroid Pulse | Plasmapheresis | 5 | 4 |
| 17 | 19 | m | NMDAR AE | Epileptic seizures, psychosis, catatonia, ataxia | WBC: 13  Protein: 665  OCBs: 3 | T2 hyperintense lesions periventricular and cerebellar with microbleeds | Serum: 1:1,000 CSF:  1:100 | Steroid Pulse | Plasmapheresis | 5 | 5 |
| 18 | 21 | f | NMDAR AE | Altered mental status, cognitive impairment | WBC: 30  Protein: 530  OCBs: 3 | Normal | Serum: 1:100 CSF:  1:100 | Steroid Pulse | Plasmapheresis | 2 | 5 |
| 19 | 24 | f | NMDAR AE | Psychosis | WBC: 201  Protein: 145  OCBs: - | Not available | Serum: 1:100 CSF:  1:3.2 | Steroid Pulse | Plasmapheresis | 5 | 5 |
| 20 | 24 | f | NMDAR AE | Psychosis, aphasia, epileptic seizures | WBC: 39  Protein: 152  OCBs: 2 | Normal | Serum: n.a. CSF:  1:100 | Steroid Pulse | Plasmapheresis | 5 | 5 |
| 21 | 62 | m | CASPR2 AE | Cognitive impairment, epileptic seizures | WBC: 3  Protein: 362  OCBs: 2 | T2 hyperintense signal both medial temporal lobes | Serum: n.a.  CSF:  1:100 | Plasmapheresis | none | 3 | 3 |
| 22 | 54 | m | CASPR2 AE | Epileptic seizures | WBC: 4  Protein: 564  OCBs: - | T2 hyperintense signal of right amygdala and hippocampus | Serum: 1:10,000 CSF: - | Steroid Pulse | none | 5 | 0 |
| 23 | 72 | m | CASPR2 AE | Cognitive impairment, epileptic seizures | WBC: 7  Protein: 397  OCBs: 3 | Nonspecific | Serum: 1:32,000 CSF:  1:1,000 | Steroid Pulse | none | 2 | 2 |
| 24 | 74 | f | CASPR2 AE | Status epilepticus, cognitive impairment | WBC: 0  Protein: 847  OCBs: 4 | Nonspecific | Serum: 1:100 CSF:  - | Steroid Pulse | none | 4 | 3 |
| 25 | 57 | m | CASPR2 AE | Cognitive impairment, Epileptic seizures, ataxia | WBC: 10  Protein: 383  OCBs: 3 | Normal | Serum: 1:10,000 CSF:  1:3,200 | Steroid Pulse | none | 2 | 2 |
| 26 | 65 | m | CASPR2 AE | Epileptic seizures, cognitive impairment | WBC: 1  Protein: 406  OCB: 1 | Increased volume of left amygdala and hippocampus | Serum: 1:3,200 CSF:  - | Steroid Pulse | none | 2 | 2 |
| 27 | 63 | m | CASPR2 AE | Epileptic seizures, cognitive impairment | WBC: 1  Protein: 420  OCBs: 4 | Normal | Serum: 1:10,000 CSF:  1:320 | Steroid Pulse | none | 2 | 2 |
| 28 | 79 | f | IgLON5 AE | Bulbar dysfunction, parasomnia,  Cognitive impairment | WBC: 4  Protein: 498  OCBs: 3 | Atrophy of mesencephalon and cerebellum | Serum: 1:1,000 CSF:  1:1,000 | Steroid Pulse | Plasmapheresis | 2 | 2 |
| 29 | 80 | m | IgLON5 AE | Bulbar dysfunction, cognitive impairment | WBC: 1  Protein: 269  OCBs: 4 | Not available | Serum: 1:1,000 CSF:  1:10 | none | none | 5 | 5 |
| 30 | 59 | m | IgLON5 AE | Bulbar dysfunction, insomnia, cognitive impairment, vertical gaze paresis | WBC: 1  Protein: 264  OCBs: 4 | Normal | Serum: 1:3,200 CSF:  1:100 | Steroid Pulse | none | 3 | 3 |
| 31 | 62 | m | GFAP AE | Myoclonic seizures, startle sign, gait instability, cognitive impairment | WBC: 79  Protein: 1862  OCBs: 3 | nonspecific | Serum: 1:1,000 CSF:  1:100 | Steroid Pulse | none | 2 | 2 |
| 32 | 76 | m | DPPX AE | Impaired consciousness | WBC: 2  Protein: 708  OCBs: - | nonspecific | Serum: 1:320 CSF:  1:1 | Steroid Pulse | Plasmapheresis | 5 | 5 |
| 33 | 73 | m | GlyR AE | Delirium, impaired consciousness, tetraparesis | WBC: 125  Protein: 1400  OCBs: 1 | nonspecific | Serum: 1:320 CSF:  - | Plasmapheresis | none | 5 | 5 |
| 34 | 48 | m | GlyR AE | Status epilepticus, altered mental status | WBC: 33  Protein: 538  OCBs: - | T2 hyperintense signal of limbic system | Serum: 1:32 CSF:  1:10 | Steroid Pulse | Plasmapheresis | 5 | 5 |
| 35 | 21 | f | GAD 65 AE | Cerebellar ataxia, epileptic seizures, cognitive impairment | WBC: 14  Protein: 204  OCBs: 3 | Increased volume and T2 hyperintense signal of left hippocampus | Serum: 1:10,000 CSF:  1:320 | Steroid Pulse | Plasmapheresis | 4 | 3 |

| **Supplementary Table 2 \| Comparison between distinct subtypes of AE** | | | | | | | | | | | |
| --- | --- | --- | --- | --- | --- | --- | --- | --- | --- | --- | --- |
|  | **Type of AE Statistical analysis (*p* value)** | | | | | | | | | | |
|  | NMDAR | LGI1 | CASPR2 | IgLON5 | NMDAR/  LGI1 | NMDAR/  CASPR2 | NMDAR/  IgLON5 | LGI1/  CASPR2 | LGI1/  IgLON5 | CASPR2/IgLON5 |  |
| n | 5 | 6 | 7 | 3 |  |  |  |  |  |  |  |
| Median age  at admission,  y (IQR) | 24  (20-29) | 78 (62-79) | 63 (57-73) | 79 (59-79) | **0.004** | **0.003** | **0.036** | 0.073 | 0.548 | 0.267 |  |
| Female,  n (%) | 4 (80) | 2 (33) | 1 (14) | 1 (33) | 0.112 | **0.023** | 0.187 | 0.461 | 1.000 | 0.490 |  |
| Median Time from onset to admission, d (IQR) | 14 (4-39) | 135 (24-338) | 120 (30-630) | 210 (180-210) | 0.082 | 0.106 | 0.095 | 0.731 | 0.643 | 0.889 |  |
| ICU admission, n (%) | 5 (100) | 0 (0) | 0 (0) | 1 (33) | **<0.001** | **<0.001** | **0.035** | 1.000 | 0.134 | 0.107 |  |
| Median total LOS, d (IQR) | 38 (33-49) | 24 (20-36) | 12 (11-21) | 28 (27-28) | 0.052 | **0.003** | 0.071 | **0.014** | 0.381 | **0.017** |  |
| Median Hospital costs, € (IQR) | 76,566 (58,183-90,228) | 24,281 (20,487-36,675) | 12,141 (11,129-21,246) | 33,387 (27,316-46,207) | **0.009** | **0.003** | 0.071 | **0.014** | 0.167 | **0.017** |  |
| Median outpatient care costs, € (IQR) | 364 (61-425) | 182 (121-334) | 243 (243-364) | 485 (364-485) | 0.792 | 0.755 | 0.143 | 0.366 | **0.048** | **0.033** |  |
| Median mRS at admission (IQR) | 5 (3.5-5) | 3 (2-4) | 2 (2-4) | 3 (2-3) | 0.621 | 0.198 | 0.673 | 0.391 | 1.000 | 0.490 |  |
| Median mRS at  discharge (IQR) | 5 (4.5-5) | 3 (2-3) | 2 (2-3) | 3 (2-3) | 0.154 | **0.013** | 0.168 | 0.170 | 1.000 | 0.260 |  |

Abbreviations: AE = autoimmune encephalitis; CASPR2 = contactin-associated protein-like-2; ICU = intensive care unit; IgLON5 = immunoglobulin-like cell adhesion molecule-5; IQR = interquartile range; LGI1 = leucine-rich glioma-inactivated-1;

mRS = modified Rankin Scale; NMDAR = N-methyl-D-aspartate receptor.

Continuous variables were compared using the Mann-Whitney U test. Categorical variables were compared using the chi-square test. For analysis, a dichotomous classification of the mRS score was performed into mildly affected (mRS ≤ 2)

and severely affected (mRS > 2). *p* Values that reached statistical significance (*p* ≤ 0.05) are highlighted in bold.
